# Evaluation of an *IDH1/2* Mutation FastTrack Assay for Patients with Cholangiocarcinoma

**DOI:** 10.3390/cancers17050820

**Published:** 2025-02-27

**Authors:** Melanie Winter, Silvana Ebner, Nina Scheuber, Falko Schulze, Maximilian N. Kinzler, Dirk Walter, Peter J. Wild

**Affiliations:** 1 Dr. Senckenberg Institutes of Pathology and Human Genetics, University Hospital Frankfurt, Goethe University Frankfurt, 60590 Frankfurt, Germany; 2 University Hospital Frankfurt MVZ GmbH, 60590 Frankfurt, Germany; 3 Medical Clinic 1, University Hospital Frankfurt, Goethe University Frankfurt, 60590 Frankfurt, Germany

**Keywords:** cholangiocarcinoma, *IDH1*, *IDH2*, Idylla™, Biocartis, Oncomine Comprehensive Assay v3 GX, Genexus, pathology, molecular

## Abstract

Background: Cholangiocarcinoma, a malignancy originating from the bile ducts, poses significant treatment challenges due to its typically late diagnosis and limited therapeutic options. However, recent advances in molecular genetics enable more personalized treatment approaches. A notable breakthrough in this context is the identification of isocitrate dehydrogenase (IDH) mutations, particularly *IDH1* and *IDH2*, which occur in a subset of cholangiocarcinoma patients. Those with *IDH1/2* mutations may benefit from targeted therapies. For instance, Ivosidenib, an IDH1 inhibitor, has shown efficacy in clinical trials, offering a new therapeutic option for patients with *IDH1*-mutant cholangiocarcinoma. Developing and implementing standardized protocols for testing and reporting mutation status are crucial for consistency and accuracy in clinical practice. Both the Idylla™ IDH1-2 Mutation Assay Kit as a FastTrack method and Next-Generation Sequencing (NGS) panels play critical roles in molecular characterization of cholangiocarcinoma. Methods: Under this aspect, a set of cholangiocarcinomas was tested using the Idylla™ platform regarding the respective recommended guidelines and standards of DIN EN ISO:17020 and DIN EN ISO:15198. Results: Overall, 25 clinically diagnosed intrahepatic cholangiocarcinomas or Adeno-CUPs were analyzed. *IDH1/2* mutations were identified in 68% (17/25) of cases using both methods, with high concordance between NGS and Idylla™ results. Discrepancies were observed in two samples, where Idylla™ detected no mutations, but NGS reported *IDH1* and *IDH2* mutations, respectively. Conclusions: Idylla^TM^ offers a rapid, user-friendly, and specific method for detecting *IDH1/2* mutations, ideal for immediate clinical needs. NGS, while more time-consuming and costly, provides comprehensive genetic profiles valuable for personalized medicine and research. The choice between these methods should be guided by the clinical context, resource availability, and individual patient needs. For routine diagnostics, we recommend an algorithmic approach starting with the FastTrack method followed by NGS for wildtype cases.

## Simple Summary

Cholangiocarcinoma, a malignancy of the bile ducts, is challenging to treat due to late diagnosis and limited options. Advances in molecular genetics enable personalized approaches, including targeting isocitrate dehydrogenase (*IDH*) mutations, found in some cholangiocarcinoma patients. IDH1 inhibitor Ivosidenib has shown efficacy in clinical trials, offering new hope for patients with *IDH1*-mutant cholangiocarcinoma. Standardized testing protocols are vital for consistent and accurate mutation detection. In this study we established the Idylla™ IDH1-2 Mutation Assay Kit and evaluated the results compared to Next-Generation Sequencing (NGS), a key tool for molecular characterization. Idylla™ provides a rapid, user-friendly method for *IDH1/2* mutation detection, suited to immediate clinical needs. Meanwhile, NGS delivers comprehensive genetic profiles, valuable for personalized medicine and research, albeit with higher cost and longer processing times. Choosing between these methods depends on clinical context, resources, and patient-specific needs.

## 1. Introduction

*IDH1* and *IDH2* mutations are significant genetic alterations found in various tumor entities, particularly in cholangiocarcinomas, gliomas, and hematological malignancies. *IDH1* mutations are commonly found in lower-grade gliomas (WHO grades II and III) and secondary glioblastomas. *IDH2* mutations occur less frequently than *IDH1* ones. *IDH1/2* mutations are also found in acute myeloid leukemia (AML), with *IDH2* mutations being more prevalent, resulting in specific clinical features and a distinct prognosis.

Cholangiocarcinoma is a rare (2–3 cases per 100,000) but aggressive cancer that develops from the cells of the bile ducts. At around 5–15%, it is the second most common liver malignancy. As people become older, the risk of CCA increases, with men being affected more often than women [1,2,3,4]. A distinction is made between intrahepatic and extrahepatic cholangiocarcinomas [5,6,7,8].

The Identification of *IDH* mutations in cholangiocarcinomas has clinical and therapeutic implications; patients can benefit from targeted therapy followed by resection through the detection of an *IDH* mutation [9]. The 5-year survival is approximately 10%, and after R0 resection it is 20–50% [10]. It has also been shown that Ivosidenib was effective in patients with *IDH1*-mutated cholangiocarcinoma [11,12]. An *IDH1/2* mutation is found in approximately 28% of intrahepatic cholangiocarcinomas and approximately 7% in extrahepatic cholangiocarcinomas [13]. *IDH1/2* play an important role in cell metabolism and are involved in the conversion of isocitrate to α-ketoglutarate. The most common mutations in the *IDH1/2* genes lead to a change in the amino acid sequence at certain positions in the protein. These mutations cause the enzyme to lose its normal function and instead perform an abnormal metabolic reaction that converts α-ketoglutarate to 2-hydroxyglutarate, which is a bona fide oncometabolite [14,15,16]. This oncometabolite may contribute to cancer development and progression by affecting various cellular processes, including cell growth, differentiation, and DNA repair. Mutations in fumarate hydratase (FH), an enzyme of the Tricarboxylic Acid (TCA) cycle, also lead to hereditary and sporadic forms of cancer that establish novel paradigms of oncometabolism [17]. In this article, we briefly review the FastTrack identification of *IDH1/2* alteration of cholangiocarcinoma.

Distinct methods exist for identifying *IDH1/2* mutations. Each method has unique features, advantages, and limitations, which can impact their clinical utility.

One of the most used methods is, for instance, Sanger sequencing, which allows the identification of specific mutations and is often used to confirm mutations detected by other methods or to analyze specific regions of interest in a targeted manner. Immunohistochemistry does not directly detect *IDH1/2* mutations; it can be used to assess the expression of downstream metabolic pathways affected by these mutations. The digital droplet PCR (ddPRC) is useful for detecting low-frequency mutations and assessing mutation burden in a sample. Next-Generation Sequencing is commonly used in comprehensive genomic profiling of tumors, allowing for the detection of *IDH1/2* mutations alongside other genetic alterations. Quantitative real-time PCR-based methods are employed for rapid detection of known *IDH1/2* mutations, particularly in cases where quick results are needed. The Idylla^TM^ IDH1-2 Mutation Assay Kit, developed by Biocartis [18], is a fully automated, real-time PCR-based platform designed for rapid and sensitive detection of *IDH1* and *IDH2* mutations [19,20]. This system is proofed as a cost-effective and rapid prescreening prior to a NGS method for routine diagnostics [21,22]. Under this aspect, a set of FFPE samples was used for the assessment of the Idylla^TM^ platform regarding the respective recommended guidelines and standards of DIN EN ISO:17020 and DIN EN ISO:15198 [23].

## 2. Materials and Methods

### 2.1. Collective of Patients

Formalin-fixed paraffin-embedded (FFPE) samples from 25 patients at the University Hospital Frankfurt/Main with a clinically diagnosed intrahepatic cholangiocarcinoma or Adeno-CUP (cancer of unknown primary) were included. All samples were previously tested using NGS and included 19 positive and 6 negative *IDH1/2* cases. A macrodissection was performed to achieve a tumor cell content (TCC) of at least 15%. Both methods were performed on the same material.

### 2.2. NGS Diagnostics

The DNA was extracted using the QIAamp^®^ DNA Micro Kit (50) (Qiagen N.V., Venlo, The Netherlands) according to the manufacturer’s instructions. For quantitative determination of the DNA, the concentration was measured using the Qubit™ 4.0 system and dsDNA HS Assay Kit according to the protocol. Seraseq^®^ Tumor Mutation DNA Mix v2 AF10 (SeraCare, Milford, MA, USA) was used for quality assurance.

For the library preparation, a panel-specific amount of 20 ng of DNA for each sample was used for the Oncomine Comprehensive Assay v3 (Thermo Fisher Scientific, Waltham, MA, USA). Library preparation was performed according to the manufacturer’s protocol. The Ion Chef^TM^ system (Thermo Fisher) was used for clonal amplification and chip loading. The subsequent sequencing was performed on the Ion GeneStudio^TM^ S5 (Thermo Fisher Scientific, Waltham, MA, USA). Data analysis was performed using the analysis software platforms provided by the manufacturer (Thermo Fisher Scientific, Waltham, MA, USA). The primary analysis of the sequencing data was completed by Torrent Suite™ software (version 5.18.1). Afterward, data were analyzed with the Ion Reporter™ software (version 5.12.0.0); filter chains Oncomine Variants 5.12 and Oncomine Extended 5.12 were used.

Genomic alterations were identified by the alignment on the reference genome hg19 (GRCh37) available at https://www.ncbi.nlm.nih.gov/ (accessed on 20 December 2024). To achieve reliable results, only alterations with fulfilled quality criteria were considered, such as allele frequency (AF) ≥5% and a coverage of at least 500× for the Ion S5™. Classification and interpretation of detected filtered and unfiltered variants of *IDH1/2* were evaluated. The variant annotation provided by the respective software was manually reviewed according to the online databases ClinVar [24] and Cosmic [25]. Other databases used for variant interpretation were gnomAD [26], OncoKB [27], dbSNP [28], and cBioPortal [29] (available online). For this study, the annotation of pathogenicity of the detected variants was determined according to the ClinVar classification in “benign”, “likely benign”, “uncertain significance”, “likely pathogenic”, and “pathogenic”. To achieve a consistent approach of naming all variants, sequence variant nomenclature was carried out in concordance with the guidelines by the Human Genome Variation Society (HGVS) [30].

### 2.3. Idylla^TM^ IDH1-2 Mutation Testing

The Idylla^TM^ IDH1-2 Mutation Assay Kit and the Idylla™ instrument (Biocartis N.V., Mechelen, Belgium) as a following automated system were applied. FFPE tissue sections or extracted DNA were placed directly on the vial solution.

Real-time PCR was performed using primers specific for the most common *IDH1* and *IDH2* mutations:

i. IDH1: p.Arg132His, p.Arg132Cyc, p.Arg132Gly, p.Arg132Ser, p.Arg132Leu;
ii. IDH2: p.Arg140Gln, p.Arg140Trp, p.Arg140Leu, p.Arg140Gly, p.Arg172Lys, p.Arg172Met, p.Arg172Gly, p.Arg172Ser, p.Arg172Trp.

Only the above variants could be detected; less common mutations such as *IDH1* p.Gly97Asp, which also produce the oncometabolite R-2-hydroxyglutarate, were missing.

The Idylla^TM^ platform uses built-in software to handle all aspects of mutation detection and reporting. It directly provides a final report, which includes:

i. Mutation status (positive/negative for *IDH1/2* mutations);
ii. Cycle threshold (Cq) values from the real-time PCR analysis.

## 3. Results

**Figure 1:**
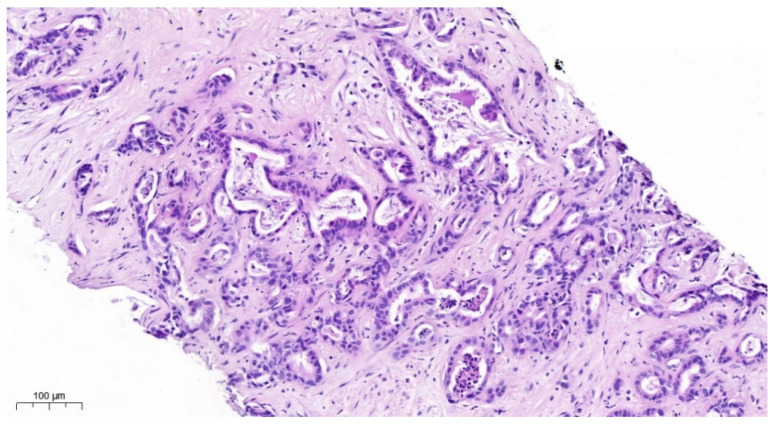
HE staining. iCCA consists of infiltrating glands in an abundant desmoplastic; fibrous stroma glands are lined by atypical cuboidal cells with varying degrees of pleomorphism intraluminal cellular debris.

Overall, 25 clinically diagnosed intrahepatic cholangiocarcinomas (Figure 1) or Adeno-CUPs of six women (24%) and 19 men (76%) at the ages of 40 to 83 years (mean: 64 years, median: 63 years) were analyzed.

### 3.1. Results Obtained by NGS as a Comparative Method

The average percentage of the tumor cell content was 44% (min = 15%, max = 80%). Among these cases, 76% (19/25) were positive for *IDH1* and *IDH2*, and 24% (6/25) were negative, as shown in Table 1. The average percentage of allele frequency (AF) of the detected mutations was 26.9% (min = 6%, max = 66%). The samples were enriched according to the presence of an *IDH1* or *IDH2* variant.

**Figure 2:**
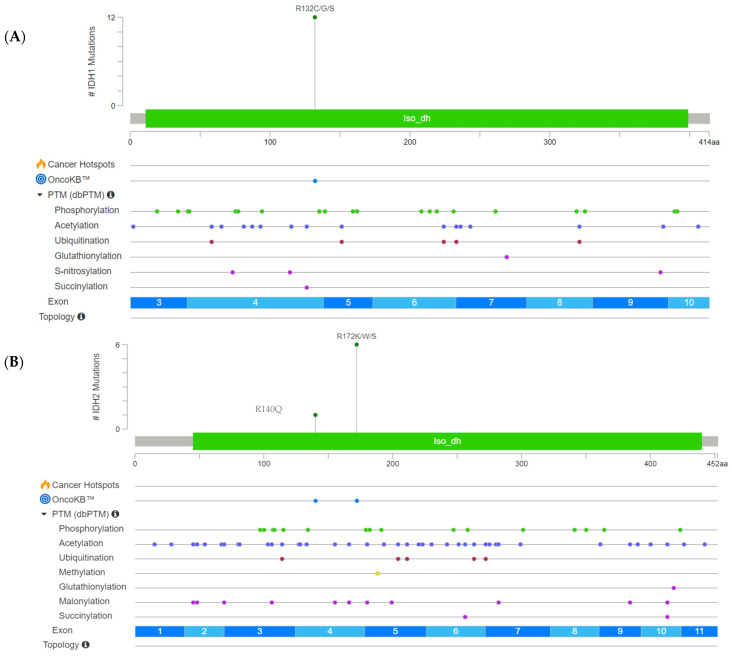
NGS-based detected *IDH1* (**A**) and *IDH2* (**B**) missense mutation types with respect to die hotspot region characterized by OncoKB and post-translational modifications (PTMs).

The detected hotspot mutations for *IDH1* were located at codon 132 (p.Arg132Cys/Gly/Ser) as visible in the lolliplot below (Figure 2A). *IDH2* mutations were detected at codon 140 (p.Arg140Gln) and codon 172 (p.Arg172Lys/Trp/Ser) (Figure 2B).

The following visualized representation, Figure 2, summarizes the *IDH1/2* mutation landscape of the set of cholangiocarcinomas and provides a comprehensive overview of the detected genetic hotspot alterations with involved exons, respectively.

### 3.2. Overall Performance of the Idylla^TM^ IDH1-2 Mutation Assay Kit

The dataset shown in Table 2 includes 25 samples with DNA concentrations ranging from 0.233 ng/μL to 35.490 ng/μL.

*IDH1/2* mutations were identified in 68% (17/25) of cases using both methods, with high concordance between NGS and Idylla™ results. Discrepancies were observed in two samples, 3 and 11, where Idylla™ detected no mutations, but NGS reported *IDH1* and *IDH2* mutations, respectively.

Median Cq values ranged from 29.7 to 35.2. Samples with lower DNA input (<0.5 ng/μL) had higher Cq values, indicating potential limitations of the Idylla™ platform in cases of low DNA yield.

Additionally, the limit of input amount was tested by a serial dilution of a positive case. A concentration of below 1 ng/µL results in a false negative evaluation.

Table 3 presents the overall concordance and Kappa Correlation Coefficient (r_(Phi)_) of Idylla^TM^ Biocartis compared to NGS.

Finally, the sensitivity for Idylla^TM^ *IDH1* and *IDH2* detection was 91.7% and 83.3%, respectively. The specificity for both genes was 100%, and the accuracy was 94.4% and 95.7% for *IDH1* and *IDH2*, respectively. The concordance of the Idylla^TM^ Biocartis system and the reference method (NGS) showed a good overall correlation coefficient of r_(Phi)_ = 0.8186 for *IDH1* and r_(Phi)_ = 0.887 for *IDH2*.

*IDH1* variants were detected in 91.7% (11/12) and *IDH2* variants in 85.7% (6/7) of previously confirmed positive cases. All *IDH1* (13/13) and *IDH2* (18/18) variants were accurately classified as wild types in alignment with previously confirmed *IDH1*- or *IDH2*-negative cases.

## 4. Discussion

Cholangiocarcinoma, or bile duct cancer, presents several unique challenges in diagnostics that are important to highlight. One of the primary clinical challenges is the often-late presentation of symptoms, which can lead to a delayed diagnosis. Patients may experience non-specific symptoms such as jaundice, weight loss, and abdominal pain, which can be mistaken for other conditions. Furthermore, the anatomical location of the bile ducts can complicate imaging and biopsy procedures, leading to challenges in obtaining accurate tissue samples for histological and molecular analysis, which underscores the importance of a multidisciplinary approach [31].

Additionally, the mutation patterns in cholangiocarcinoma can differ significantly from other cancers. For instance, mutations in the *IDH1* and *IDH2* genes, as well as alterations in the *FGFR2* gene, are more commonly associated with intrahepatic cholangiocarcinoma. These specific mutation patterns can influence treatment options and prognostic outcomes, making genetic testing a crucial component of the diagnostic process [32].

The identification of cholangiocarcinoma patients with *IDH1/IDH2* variants, which are mutually exclusive, may benefit from targeted therapies. Thus Ivosidenib, an IDH1 inhibitor, provides a new therapeutic option for patients with *IDH1*-mutant cholangiocarcinoma.

The Idylla^TM^ Biocartis system was evaluated under real-world conditions using FFPE samples or the corresponding extracted DNA. Compared to NGS diagnostics, it offers a significantly shorter turnaround time and utilizes a faster prescreening technology. In this project, the Idylla^TM^ *IDH1-2* Mutation Assay Kit allowed the detection of 89.5% *IDH1/2* (17/19) mutations of previously tested positive cases. In a comparative study by James P. Solomon’s Department of Pathology and Laboratory Medicine (Weill Cornell Medicine, New York), 39 samples from glioma patients treated on the New York-Presbyterian Hospital (NYPH) campus between 2018 and 2023 were examined regarding *IDH1/2* status.

The prevalence of *IDH* mutations varies significantly between gliomas and cholangiocarcinomas. In gliomas, particularly in lower-grade gliomas and secondary glioblastomas, *IDH* mutations are quite common, with prevalence rates ranging from 40% to 80%, depending on the specific subtype. In contrast, cholangiocarcinomas, which are cancers of the bile ducts, have a lower prevalence of *IDH* mutations, typically around 10–20%.

In terms of performance differences, *IDH* mutations in gliomas are associated with distinct clinical and pathological features, often correlating with a better prognosis compared to *IDH*-wildtype tumors. In cholangiocarcinoma, *IDH* mutations can also influence the tumor’s behavior and response to treatment, but the overall impact on prognosis is less well defined compared to gliomas [33]. The results of the mentioned study of the two assays showed a concordance of 97% (38/39 samples) and an LOD of AF of 2.5–5% [34]. The results of the study largely correspond to the results of this work, which provides an overall concordance of 92% (23/25) regarding negative and positive *IDH1/2* samples. After serial dilution, the detection limit with respect to the initial concentration was 1 ng/μL. Poor DNA quality can also interfere with the detection of a mutation, which can cause the detection limit to fluctuate.

The two discordant negative results can also be explained by the limited DNA quantity or quality. For example, in one case, the DNA concentration was finally 0.2 ng/µL, after diluting with water up to the required volume of 50 µL. This sample had the lowest DNA concentration in the entire collective. Unfortunately, it was not possible to confirm these discrepancies by repetition or another method due the limited material available. The quality of the DNA extracted from the samples can significantly impact the results. Generally, other possible reasons for obtaining different results may be due different methodological parameters, for instance, in the detection limits or primer sets. Some mutations may exist in subclonal populations within the tumor. If one method is more sensitive to detecting subclonal mutations, it may report a mutation that another method does not. In summary, discordant mutation status results from two different methods can arise from a combination of methodological differences, sample quality, technical errors, biological factors, and interpretation issues. While *IDH* mutations are less common in cholangiocarcinoma compared to gliomas, their presence can influence treatment decisions and prognostic assessments. A false negative result may lead to a misleading prognosis underestimating the aggressiveness of the tumor. A false negative result may lead to inadequate follow-up or surveillance strategies, increasing the risk of undetected recurrence. IDH mutations are often associated with specific tumor characteristics and may indicate a different biological behavior. If a patient is incorrectly classified as IDH-wildtype, they may not receive appropriate monitoring or treatment resulting in missed opportunities for targeted therapy, potentially affecting patient outcomes. Additionally, many clinical trials are designed to include patients with specific genetic mutations. A false negative IDH status could exclude patients from potentially beneficial clinical trials that target IDH-mutant cholangiocarcinoma. To resolve these discrepancies and avoid false negative results, we recommend an algorithmic approach for routine diagnostics: prescreening with the FastTrack method, and in the case of a wildtype result, follow with NGS.

As already mentioned, the fully automated analysis takes around 90 min, is quite user-friendly, and is suitable for routine application in molecular pathology. However, mutation coverage is limited to specific hotspot mutations, and it offers less flexibility in terms of customizing or expanding the testing range. With the DNA cartridge and the specific vial for *IDH1/2* mutation analysis, five *IDH1* mutations in codon R132, four *IDH2* mutations in codon R140, and six *IDH2* mutations in codon R172 can be detected. A maximum of eight instruments can be controlled with one Idylla^TM^ console, allowing up to eight analyses to be run simultaneously. The Idylla^TM^ platform is particularly appropriate for smaller laboratories; the instruments are suitable for stacking and therefore fit on small tables. The risk of contamination is very low even when using several instruments at the same time, as each cartridge represents a closed system. The cost of running a sample is around €100–200. Contrarily, Next-Generation Sequencing is more expensive but allows a comprehensive mutational analysis of several hundreds of genes simultaneously, including *IDH1* and *IDH2*. NGS enables the detection of mutations, copy number variations, and gene fusions in a single test in parallel. The sensitivity is also high, meaning that rare mutations at low allele frequencies can also be detected. The disadvantages of this system are the higher complexity, which requires specialized expertise and extensive data analysis, as well as the longer turnaround time, which takes several days from sample preparation to result. In addition, the costs are higher than with PCR-based methods due to the complexity and the required infrastructure. Furthermore, there is a significantly higher risk of contamination here than with the Idylla^TM^ platform, due to the many intermediate steps of manual work when preparing several samples at the same time.

## 5. Conclusions

In conclusion, the Idylla^TM^ platform is a rapid method and is particularly suitable as a pre-screening method for mutations.

It can be easily implemented in pathology laboratories, because it is a fully automated test, not requiring DNA extraction, and easy to interpret. Table 4 shows the comparison of the Idylla™ and NGS workflow, emphasizing differences in turnaround time, complexity, and costs.

The Idylla™ workflow could be integrated into the diagnostic workflow in case of urgent molecular analysis for cholangiocarcinoma patients who require a rapid therapeutic decision, or when a platform for multiplex DNA analysis is not available. Highly complex tests such as NGS can make it difficult for clinicians to enroll their patients in a clinical trial in a timely manner. The repetition of this specific Idylla^TM^ assay needs sufficient material, which is why this procedure should be limited to emergency cases or when NGS analysis is not possible within ten working days or fails. The absence of an alteration requires additional testing by an orthogonal NGS-based method. Additionally, in order to provide detailed information about the mutation, a subsequent test using an NGS method would be suitable.

The choice of method for detecting *IDH1* and *IDH2* mutations in clinical practice depends on various factors, including the specific clinical context, the need for sensitivity and specificity, available resources, and the type of samples being analyzed. Often, a combination of these methods may be employed to ensure accurate and reliable detection of mutations, guiding treatment decisions and patient management.

For routine *IDH1/2* diagnostics, we recommend an algorithmic approach starting with the FastTrack method followed by NGS for wildtype cases.

## Figures and Tables

**Table 1:** Patient collective with respect to demographic features as well as the *IDH1/2* mutation status determined with NGS. F = female, M = male, TCC = tumor cell content, AA = amino acid, AF = allele frequency.

Study-No.	Gender	Age	TCC [%]	*IDH1/2* Status	Nucleotide Substitution	AA Substitution	AF [%]
1	F	57	15	*IDH1*	c.394C>T	p.Arg132Cys	15.40
2	M	74	15–20	*IDH1*	c.394C>T	p.Arg132Cys	15.95
3	F	58	20–25	*IDH1*	c.394C>T	p.Arg132Cys	17.70
4	F	58	40	*IDH1*	c.394C>T	p.Arg132Cys	33.03
5	M	40	30	*IDH1*	c.394C>G	p.Arg132Gly	6.00
6	M	60	60	*IDH1*	c.394C>T	p.Arg132Cys	17.68
7	M	58	20	*IDH1*	c.394C>T	p.Arg132Cys	9.79
8	M	69	70	*IDH1*	c.394C>A	p.Arg132Ser	54.10
9	M	58	30	*IDH2*	c.514A>T	p.Arg172Trp	66.00
10	M	63	50	*IDH2*	c.514A>T	p.Arg172Trp	21.40
11	M	53	60	*IDH2*	c.515G>A	p.Arg172Lys	32.10
12	M	76	15	Negative	--	--	--
13	M	82	50	Negative	--	--	--
14	M	60	80	Negative	--	--	--
15	F	58	80	Negative	--	--	--
16	M	71	30	Negative	--	--	--
17	M	83	70	Negative	--	--	--
18	M	67	70	*IDH1*	c.394C>T	p.Arg132Cys	30.50
19	M	75	40	*IDH1*	c.394C>T	p.Arg132Cys	22.22
20	M	63	20	*IDH1*	c.394C>T	p.Arg132Cys	19.61
21	F	57	20	*IDH1*	c.394C>T	p.Arg132Cys	32.99
22	M	53	75	*IDH2*	c.515G>A	p.Arg172Lys	52.03
23	F	63	45	*IDH2*	c.516G>C	p.Arg172Ser	20.26
24	M	78	60	*IDH2*	c.515G>A	p.Arg172Lys	36.85
25	M	66	40	*IDH2*	c.419G>A	p.Arg140Gln	8.17

**Table 2:** Patient collective with respect to the *IDH1/2* mutation status (NGS vs. Idylla^TM^). Median Cq from internal control. * = elevated; median Cq indicates low DNA input or quality.

Study-No.	DNA (ng/μL)	NGS	Idylla^TM^ Biocartis	Codon	Median Cq
1	1.114	*IDH1*	*IDH1*	132	32.4
2	0.406	*IDH1*	*IDH1*	132	33.9
3	0.233	*IDH1*	negative	--	35.2 *
4	1.629	*IDH1*	*IDH1*	132	31.6
5	1.125	*IDH1*	*IDH1*	132	33.4
6	0.464	*IDH1*	*IDH1*	132	34.0
7	0.442	*IDH1*	*IDH1*	132	34.1
8	8.426	*IDH1*	*IDH1*	132	31.2
9	1.278	*IDH2*	*IDH2*	172	32.9
10	35.490	*IDH2*	*IDH2*	172	30.0
11	21.500	*IDH2*	negative	--	31.2
12	2.145	negative	negative	--	34.8
13	2.336	negative	negative	--	32.5
14	1.804	negative	negative	--	34.6
15	9.804	negative	negative	--	32.1
16	2.801	negative	negative	--	33.3
17	14.586	negative	negative	--	30.5
18	0.663	*IDH1*	*IDH1*	132	35.0
19	3.503	*IDH1*	*IDH1*	132	32.2
20	0.519	*IDH1*	*IDH1*	132	34.1
21	0.493	*IDH1*	*IDH1*	132	35.2 *
22	26.660	*IDH2*	*IDH2* *IDH2*	140 172	29.7
23	0.486	*IDH2*	*IDH2*	172	35.1 *
24	8.540	*IDH2*	*IDH2*	172	32.0
25	2.212	*IDH2*	*IDH2*	140	32.7

**Table 3:** Concordance analysis for *IDH1* and *IDH2*.

Idylla^TM^ vs. NGS	*IDH1*	*IDH2*
Sensitivity	91.7%	83.3%
Specificity	100%	100%
Accuracy	94.4%	95.7%
Correlation coefficient r_(Phi)_	0.8186	0.887
Concordance		
Right positive	91.7%	85.7%
Right negative	100%	100%

r_(Phi)_: The value of r ranges between −1 and 1. A correlation of −1 shows a perfect negative correlation, while a correlation of 1 shows a perfect positive correlation. A correlation of 0 shows no relationship between the movement of the two variables.

**Table 4:** Comparison of the Idylla™ and NGS workflow.

	Idylla^TM^ Biocartis	NGS
Technology	quantitaive real-time PCR	sequencing
Gene coverage	limited	95%
Time required	<2 h	several days
Automation	fully automated	depending on the platform
Risk of cross contamination	low, closed system	medium to high
Cost/sample	low	high
User-friendliness	high, easy to use	technical expertise required

## Data Availability

Data are contained within the article.
